# Limiting parental feedback disrupts vocal development in marmoset monkeys

**DOI:** 10.1038/ncomms14046

**Published:** 2017-01-16

**Authors:** Yasemin B. Gultekin, Steffen R. Hage

**Affiliations:** 1 Neurobiology of Vocal Communication, Werner Reichardt Centre for Integrative Neuroscience, University of Tübingen, Otfried-Müller-Str. 25, Tübingen 72076, Germany

## Abstract

Vocalizations of human infants undergo dramatic changes across the first year by becoming increasingly mature and speech-like. Human vocal development is partially dependent on learning by imitation through social feedback between infants and caregivers. Recent studies revealed similar developmental processes being influenced by parental feedback in marmoset monkeys for apparently innate vocalizations. Marmosets produce infant-specific vocalizations that disappear after the first postnatal months. However, it is yet unclear whether parental feedback is an obligate requirement for proper vocal development. Using quantitative measures to compare call parameters and vocal sequence structure we show that, in contrast to normally raised marmosets, marmosets that were separated from parents after the third postnatal month still produced infant-specific vocal behaviour at subadult stages. These findings suggest a significant role of social feedback on primate vocal development until the subadult stages and further show that marmoset monkeys are a compelling model system for early human vocal development.

## Abstract (web summary)

The development of mature vocal patterns is shaped by parental influence in many animals. Here, Gultekin and Hage show that parental feedback not only influences vocal development, but is indeed necessary for juvenile marmosets to acquire normal vocal behaviour.

Human vocal development is driven by learning and constrained by maturation^1^. Through endogenous vocal exploration at a very early stage^2^, infants start using genetically predetermined, prelinguistic, speech-like vocalizations (so-called protophones), and non-speech-like vocalizations (such as vegetative sounds, crying and moaning)^3^,^4^,^5^,^6^,^7^. These utterances are followed by vocalizations that consist of continuous or interrupted phonations, also called babbling^8^,^9^. Babbling becomes increasingly speech-like during the first year^3^,^10^. Direct interaction between infants and their caregivers is one of the critical elements during vocal development^1^,^11^. Some changes in the infant vocal behaviour towards speech are correlated with the auditory feedback infants receive from their caregivers^1^,^12^,^13^. As an example, it has been shown that contingency of the responses by the caregivers shape babbling and has an impact on the vocal development of infants^12^. Recent studies revealed similar developmental processes in marmoset monkeys, a highly vocal and cooperative breeding species^14^,^15^, and suggest it as a compelling model system for such early vocal development in humans^16^,^17^.

During vocal development, several bird and mammalian species, including both vocal learners and non-learners^18^,^19^,^20^,^21^, may widely undergo distinct changes in vocal behaviour, which have yet not been studied in depth across species. For example, during vocal development, marmoset vocalizations undergo distinct changes in several call parameters, such as decreases in call frequency and entropy and increases in call duration^16^,^22^. Furthermore, inter-call intervals change significantly. This change is mainly characterized by an extinction of inter-call intervals between 200 and 800 ms, as they are typically present within ‘babbling' sequences in infant marmosets that contain both immature and mature call types^17^. Additionally, contingent parental feedback accelerates vocal development into mature vocalizations in marmosets, as in humans^16^, even though the marmoset vocalizations are considered innate.

It is still unclear whether parental feedback in mammals is, as in humans, an obligate requirement for proper vocal development or whether parental feedback simply accelerates vocal development, without a detrimental effect if absent. We address this question by comparing vocal behaviour of marmoset monkeys in the subadult stage. We used two sets of offspring from the same parents: one set was normally raised, while the other was separated from the parents after the third postnatal month. Using quantitative measures to compare distinct call parameters and vocal sequence structure of the litters, we show that parental feedback is necessary for normal vocal development in marmosets.

We find that vocalizations of monkeys with limited parental feedback dramatically differ from vocalizations of their normally raised siblings. All monkeys produced mature vocalizations. However, in contrast to normally raised monkeys, marmosets with limited parental feedback also produced infant-specific vocal behaviour, which is characterized by specific call types, distributions and transitions. The vocalizations of the litters also differed in distinct call parameters, such as call frequency, duration, entropy and inter-call intervals.

## Results

### Marmoset vocal development depends on parental feedback

**Figure 1:**
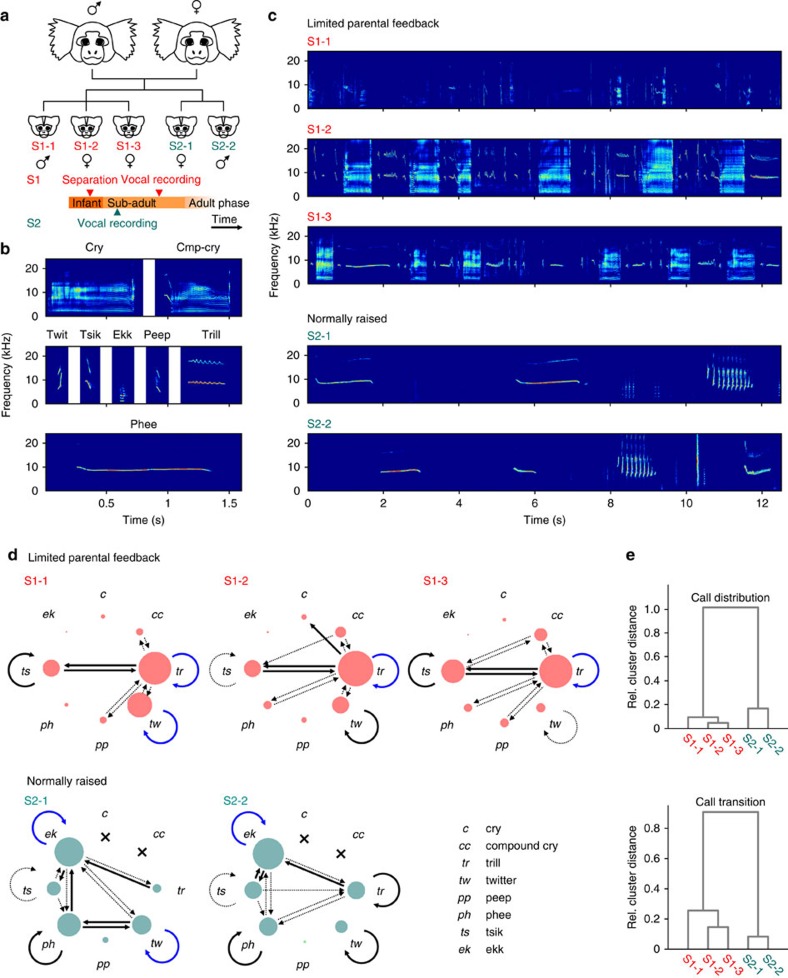
Vocal development of subadult marmoset monkeys depends on parental feedback. (**a**) Top: family relationship of the five siblings. The triplets with limited parental feedback (S1-1, S1-2 and S1-3) were born prior to the normally raised twins (S2-1, S2-2). Bottom: experimental timeline indicating separation of the S1 siblings and vocal recordings in the S1 and S2 group, respectively. (**b**) Examples of uttered call types. (**c**) Spectrograms of vocal sequences produced by the five marmoset monkeys indicate differences in vocal behaviour between S1 and S2 offspring. (**d**) Transition diagrams visualizing vocal sequences of S1 and S2 monkeys. Each node in the diagram corresponds to a type of call, and the arrows correspond to the transitions between call types. Node size is proportional to the fraction of the call types, and edge size is proportional to the transition probability between calls. Blue arrows are transitions above 10% occurrences, black solid arrows between 5% and 10% and dashed arrows between 1% and 5%. (*n*=13,582 vocalizations) (**e**) Clustering dendrogram of the five monkeys based on call distribution and transition (weighted linkage method with Spearman's proximity matrix).

We recorded 13,582 vocalizations of five subadult marmoset monkeys in a directed context, that is, with visual and auditory contact with potential caregivers. The subjects were born from the same parents in two different litters (triplets and twins) (Fig. 1a). As marmoset monkeys typically give birth to dizygotic twins^23^, siblings from our two litters are genetically as similar as same-litter siblings, making genetic differences between groups a less likely explanation. The first litter (S1) consisted of three animals that had direct contact with their parents within the first 3 postnatal months. One of these was a male (S1-1), which was hand-raised by an animal caretaker and that had several hours of daily visual and auditory contact with its parents, and two siblings (S1-2 and S1-3), which were separated from the parents at the age of 3 months in order to produce a stable social group with the hand-raised sibling in a separate cage within the same animal facility. Therefore, the S1 litter had direct contact with their parents within the first 3 postnatal months but were lacking direct parental feedback and visual contact to other conspecifics from the third postnatal month on (limited parental feedback siblings). The second litter (S2) consisted of a female (S2-1) and a male (S2-2) and was still grouped with the parents at the time of the vocal recordings (normally raised siblings). Therefore, both litters were held in similar conditions, which only differed in the presence or lack of direct parental feedback after 3 months.

During vocal recordings, the subjects were isolated in a small cage and were in direct visual and auditory contact with potential caregivers (monkey S1-1 with an animal care-taker, all other monkeys with a pair of adult marmoset monkeys). Normally, under these conditions, subadult and adult monkeys exhibit adult call repertoire, whereas infants produce infant-specific call strings, so-called ‘babbling', that is characterized by the utterance of both immature and mature call types with short inter-call intervals (200–800 ms)^24^,^25^. In the given situation, all five monkeys, including the hand-raised sibling, produced a variety of adult call types (Fig. 1b). However, while normally raised monkeys exclusively produced adult call types, such as trills, twitter, phees, peeps, tsiks and ekks by the age of 7 months, monkeys that had limited parental feedback still produced infant vocalizations, such as cries and compound cries (Fig. 1c), at the age of 13 months. In addition, limited parental feedback monkeys also still exhibited the infant-specific ‘babbling' behaviour.

We quantified the vocal behaviour by comparing call distributions and the order of the uttered calls within the recording sessions (call transitions). Figure 1d shows differences in vocal behaviour between monkeys from litters S1 and S2. Size of nodes represents the proportion of call types, and the thickness and colour of the arrow line represents the transition probability^17^. Distribution of call types was significantly different between the five siblings (*P*<0.001, χ^2^=7,957.3, df=28, *n*=8 call types, 5 animals, *n*=13,582 vocalizations, chi-square test). S1 siblings still produced cries and compound cries, which were absent in S2 siblings. In addition, S1 monkeys still produced a large number of trill calls (>60%), mostly while ‘babbling'. S2 monkeys produced a wide range of call types, such as phees, ekks, twitter, tsiks and trills (Fig. 1d). In contrast to the S1 monkeys, however, the occurrence of trills was generally very low. The observed similarities in call-type distributions of animals in between litters were confirmed by a *post hoc* cluster analysis, which clustered S1 and S2 monkeys, respectively, together, showing that animals were more similar within one litter rather than between litters (Fig. 1e).

Furthermore, we observed significant differences in call transitions between subjects (*P*<0.001, χ^2^=9,493,9, df=240, 61 occurred types of call transitions, 5 animals, *n*=13,582 vocalizations, chi-square test), indicating high variability in vocal behaviour across animals. Overall, however, the two sets of siblings differed in their call transitions and distributions but were matched along these factors within their own litters. In S1 monkeys, trills alternated with tsiks and compound cries among several other call transitions. In contrast, vocal behaviour in S2 monkeys was predominantly characterized, among other call transitions, by ekks alternated with tsiks, phees and trills as well as consecutively produced phee and ekk calls (Fig. 1d). The observed similarities in call transition distributions of monkeys within litters were confirmed by a *post hoc* cluster analysis, which clustered S1 and S2 monkeys, respectively, together, showing that animals were more similar within a litter than between litters (Fig. 1e).

### Differences in call parameters between S1 and S2 siblings

**Figure 2:**
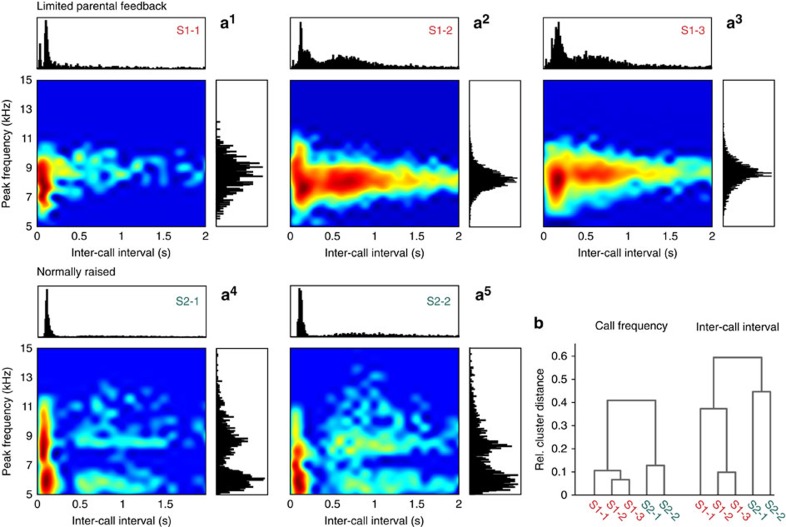
Differences in peak frequency and inter-call intervals between S1 and S2 monkeys. (**a**) Logarithmic probability maps of peak frequency and inter-call interval for S1 (**a**^1^–**a**^3^) and S2 siblings (**a**^4^ and **a**^5^). Warmer colours in the heat maps indicate higher probabilities (lower left panel in **a**^1^–**a**^5^). Histograms show absolute distribution of peak frequencies (right panel in **a**^1^–**a**^5^) and inter-call intervals (upper panel in **a**^1^–**a**^5^). (*n*=13,582 vocalization for peak frequencies and *n*=11,370 vocalizations for inter-call intervals) (**b**) Clustering dendrogram of the five monkeys based on call frequency and inter-call interval (weighted linkage method with Spearman's proximity matrix).

As a next step, we analysed acoustic parameters, such as call frequency, entropy and duration, that are known to undergo distinct changes during vocal development of monkeys^16^,^17^,^22^. Figure 2a shows the distribution of peak frequencies and inter-call intervals of the limited parental feedback monkeys and their normally raised siblings. Although call frequencies clustered between 8 and 9 kHz in S1 monkeys, in S2 monkeys they showed a bimodal distribution, with peaks between 5.5 and 6.5 kHz and between 8 and 9 kHz. For quantitative analyses on inter-call intervals, we focussed on the time range between 0 and 2 s, which corresponds to >80% of all inter-call intervals. Inter-call intervals in S1 monkeys were characterized by a bimodal distribution (Fig. 2a). The first peak between 0 and 200 ms corresponds to intervals between the same type of utterance (‘syllables') within one call, such as twitter calls, or call combinations that are uttered with short latency, such as tsik-ekks. The second peak between 200 and 800 ms corresponds to the interval between calls during the ‘babbling' sequences^17^. In contrast to S1 monkeys, distributions of inter-call intervals in S2 monkeys did not show the second peak owing to the absence of ‘babbling' behaviour. These observed factors were significantly different between monkeys in both peak frequencies (*P*<0.001, 5 animals, *n*=13,582 vocalizations, Kruskal–Wallis test) and inter-call intervals (*P*<0.001, 5 animals, *n*=11,370 vocalizations, Kruskal–Wallis test). Again, *post hoc* cluster analyses confirmed the observed differences between litters and clustered S1 and S2 siblings in their respective litter in both peak frequencies and inter-call intervals (Fig. 2b).

**Figure 3:**
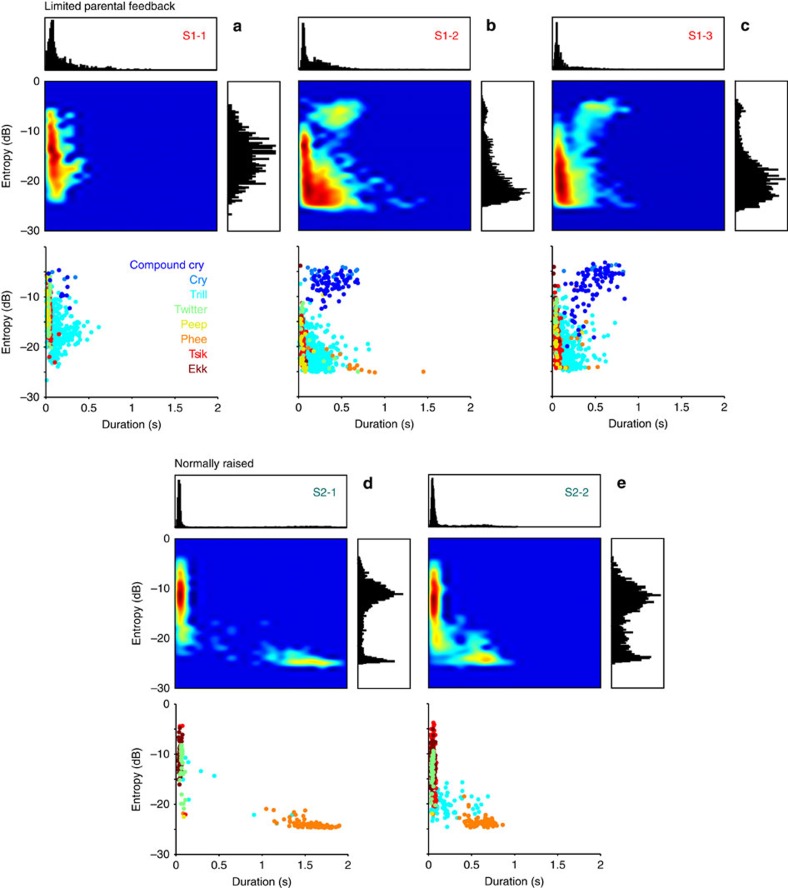
Differences in call entropy and duration between S1 and S2 monkeys. Heat maps depict logarithmic probability maps of call entropy and duration for S1 (**a**–**c**) and S2 siblings (**d**,**e**; middle left panels). Warmer colours indicate higher probabilities. Histograms show absolute distribution of call entropy (middle right panels) and duration (upper panels). Raster plots show correspondence between clusters and call types for a single recording session each (lower panels).

Finally, we evaluated distributions of call entropy and duration as a measure of the developmental stage of the underlying vocal behaviour^16^,^22^. Duration and entropy identified rather disjoint clusters in the S1 offspring. A distinct cluster with high entropy values and relatively long durations consisted of cries and compound cries that were exclusively uttered by monkeys with limited parental feedback (Figs 3a–c). Another cluster with very short durations (<0.1 s) contained mainly peeps, ekks and tsiks. A third, relatively big cluster with low entropy values and relatively long durations was mostly composed of trill calls. In contrast, normally raised S2 monkeys had two distinct clusters containing solely mature call types. Similarly to the S1 monkeys, one cluster with very short durations (<0.1 s) predominantly consisted of tsiks and ekks (Fig. 3d,e). The second cluster with low entropy values and long durations (for S1 with a mean duration of 1.5 s, for S2-2 with a mean duration of 0.7 s) exclusively contained phee calls. Overall, our cluster analyses demonstrate that the S1 siblings showed a higher degree of similarity within their litter than the two normally raised monkeys did, in the distribution of all but one tested call parameter.

These results indicate that limited parental feedback monkeys show infant-specific distributions of call parameters, such as higher peak frequencies and shorter durations compared with the normally raised monkeys, in addition to babbling behaviour. This is of particular interest, as both S2 monkeys have a lower weight than the S1 monkeys (S1: 430, 380, 400 g, respectively, versus S2: 290 g each). Therefore, differences in overall growth, which correlated well with the size of the vocal apparatus (call frequency) and lung volume (call duration) cannot account for the observed differences^26^.

## Discussion

We show that vocal behaviour of marmoset monkeys with limited parental feedback dramatically differs from vocal output of their normally raised siblings. This suggests a significant role of social feedback on primate vocal development. Our findings point to infant-specific vocal behaviour in subadult marmosets with limited parental feedback. All five monkeys produced a variety of mature call types. These findings are in accordance with previous evidence suggesting that the basic acoustic call structure is innate and does not have to be learned through auditory or social feedback^27^,^28^,^29^,^30^. In the current study, however, in contrast to the normally raised subadult marmosets that exclusively produced adult vocalizations at the age of 7 months, subadult monkeys with limited parental feedback still produced infant ‘babbling' sequences that contained both immature vocalizations, such as cries and compound cries, as well as mature call types, much later at the age of 13 months. Such babbling behaviour is typically seen in infant marmosets that normally disappears completely after the first postnatal months and does not come back in the subadult or adult phase^16^,^17^,^22^,^24^. Interestingly, vocal behaviour of the hand-raised monkey was quite similar to vocal output observed in its two siblings from the same litter. These findings suggest that the differences in vocal output between the two litters are mainly due to developmental changes after the third postnatal month rather than to the different ways vocal behaviour was elicited in the hand-raised monkey and its siblings.

Besides infant ‘babbling' sequences and immature call types, such as cries and compound cries, S1 monkeys still produced a large number of trill calls (>60%), mostly while ‘babbling', as it has been reported for marmoset infants before^25^. The vocalizations of the litters also differed in distinct call parameters. Mean call frequencies and entropies were higher, and as such more similar to infant vocalizations^17^,^22^, in animals with limited parental feedback, than in normally raised marmosets. In addition, inter-call intervals were significantly lower in monkeys with limited parental feedback than the S2 monkeys. This difference is mainly caused by an extinction of inter-call intervals between 200 and 800 ms, as they are typically present within infant ‘babbling' sequences^17^.

Recent studies thoroughly investigated the vocal development of marmoset monkeys in the first postnatal weeks^16^,^17^ and revealed a direct impact of parental feedback on the transition from infant vocalizations to adult call types^16^. Our results suggest a crucial role of parental feedback on vocal development beyond infancy. Parental feedback seems to be critical to the pruning of infant vocalizations and shaping of the vocal repertoire of young marmosets into the subadult phase.

However, limited parental vocal feedback might not be the only reason for such changes in vocal behaviour of young marmosets separated from their parents at an early age. Confounding aspects, such as environmental and psychological factors, might have caused the observed vocal abnormalities in these animals. First of all, separation of the subadult siblings from the parents was initially motivated by a chance to establish a stable social group with the hand-raised monkey instead of housing it alone. We attempted to minimize environmental differences by moving the S1 siblings into a cage with a largely identical layout and within the same room as the parental cage (see Methods section for details). Furthermore, we paid particular attention to find the best separation time to reunite the S1 siblings. On one hand, we wanted to ensure that the siblings stayed long enough with their parents in order to minimize potential psychological confounds, such as an elongation of immaturity (with an accompanied continuation of infant vocalizations). On the other hand, to increase the likelihood of successful reunification, it was important to reunite the three siblings at an early age. For the following reasons, we decided to reunite the siblings after the third month. After this period, weaning is largely completed and young marmosets already spend most of their time away from their parents and locomote independently^31^,^32^,^33^. These findings were supported by our personal observations during the last 2 weeks prior to separation, showing that the siblings were moving around independently and were barely being carried by their parents. In addition, they were able to independently eat out of food dishes and to autonomously hunt small offered prey (for example, mealworms and locusts). Immediately after reunification, we observed no aggressive behaviour. Instead, we witnessed affiliative interactions, such as grooming behaviour, between the S1 siblings. This continued throughout the following months indicating a healthy, stable social group^34^. Abnormal or atypical behaviour other than the reported vocal differences has not been observed. In this context, it is important to note that the hand-raised monkey and the two siblings spent different amounts of time with their parents prior to the separation and thus might have had different psychological experiences during this time. However, the vocal behaviour was more similar within the S1 litter than it was between the two normally raised monkeys. Therefore, we assume that psychological and environmental factors might have had only minor effects on the vocal behaviour of the S1 siblings and that the observed vocal differences between groups were correlated to differences in direct parental feedback rather than other factors. Finally, we collected a large number of vocalizations to obtain a reliable data set for statistical analyses, thus compensating for the relatively small sample size in comparison to most studies on non-primate species. In addition, the control group litter was from the same parents as the S1 subjects. This approach provided the opportunity to compare monkeys with equal genetic relatedness and to largely exclude genetic variation as a main confounding factor for differences in vocal behaviour between litters.

During babbling, human infants produce sounds that resemble a maturing speech capacity, suggesting this behaviour to be an important precursor for speech^3^. Babbling behaviour is not unique to humans and can be found in a few other lineages of vocal learners, such as songbirds^35^,^36^. Zebra finch males, for example, start their vocal development with innate vocalizations. As they start learning the motifs of their individualized songs through social feedback mechanisms, they form a subsong that is comparable to human babbling. By mechanisms of contingent feedback and self-monitoring, these subsongs eventually change into mature bird songs and crystallize^37^. These findings suggest that in song birds^38^,^39^,^40^, as in human infants^3^,^35^, social guidance appears to have an essential impact on proper vocal development. In contrast, the acoustic structure of monkey vocalizations is largely innate and monkeys seem unable to learn new structures through auditory feedback^27^,^28^,^29^,^30^. However, our data indicate that social experience appears to have, as in humans, a direct effect on shaping the vocal repertoire of marmosets in the subadult phase. So, which vocal learning processes might be involved in the observed vocal behaviour?

Within the framework of vocal communication systems, three types of learning are possible: auditory comprehension learning, vocal usage learning, and vocal production learning^41^,^42^,^43^,^44^,^45^,^46^,^47^. Auditory comprehension learning is characterized by the ability to associate a distinct auditory cue with an adequate behavioural response or objects in the environment and is broadly distributed among vertebrates, including primates^41^,^42^. Vocal usage learning is defined by the ability to volitionally control when and where, but not how, to produce a specific vocalization in a specific cognitive, social or environmental context^41^. Conceptually, one might distinguish two types of vocal usage learning. First is the ability to withhold or initiate a specific vocalization, although it is still tied to the respective (motivational) context^48^,^49^,^50^. Second is the more elaborate ability to decouple calls of the monkeys' innate vocal repertoire from the accompanying motivational state for use in a novel context^29^,^30^. For example, instrumentalizing their vocal output to perform a specific task successfully in operant conditioning tasks^51^,^52^,^53^,^54^. In both types of vocal usage learning, vocal output does not have to depend on vocal imitation. Despite this, these abilities might have been one of the critical preadaptation during the evolution of human speech in the primate lineage.

However, human speech requires much more than just concatenating calls from a preexisting set of innate vocalizations, which is the ability to generate new vocal patterns, that is, vocal production learning^44^. Besides humans, vocal production learning has been found in songbirds, parrots, cetaceans, bats and a few other lineages^46^,^52^,^55^,^56^. From our current knowledge, such vocal pattern learning mechanisms do not seem to exist in non-human primates, that is, they appear to lack the ability to learn or imitate new vocal signals^29^,^44^,^57^. In the present study, we find evidence for vocal usage learning in marmosets but not for vocal production learning. Although the animals with limited parental feedback were still producing infant call types, both litters exhibited the adult call repertoire. Therefore, our findings indicate that direct auditory feedback from a caregiver can prune repertoires of innate call types and thus that vocal usage learning requires social experience in marmoset monkeys. The need of such experience for vocal development in monkeys was predicted in earlier work^48^,^49^, but, to the knowledge of the authors, has never been experimentally demonstrated. Future studies will now have to decipher whether direct parental feedback is actually capable of teaching, or rather shaping, the change in vocal behaviour of young marmoset monkeys.

From a neurophysiological perspective, our findings indicate that vocal pattern generating networks might be directly modulated during vocal development by social experience. Recently, it has been shown that monkeys are able to show vocal usage learning in a well-controlled experimental design^53^ and that prefrontal and premotor structures might serve as potential hubs controlling such complex audio-vocal communicative behaviours^58^,^59^,^60^,^61^,^62^. It would be interesting to see whether and how such frontal cortex circuits contribute to priming of vocal behaviour in response to social experience. The marmoset monkey, a highly social and loquacious species, is a compelling model system to investigate the neurophysiological principles of such audio-vocal feedback mechanisms underlying early vocal development in humans.

## Methods

### Experimental animals

We used five captive marmoset monkeys (*Callithrix jacchus*): one set of 13-month-old subadult triplets (S1) weighting 430, 380, 400 g, respectively, and one set of 7-month-old subadult twins (S2) weighting 290 g each. They were born in two different litters to the same parents. Differences in their weight are well correlated with their difference in age, the adult weight of their parents and within the weight range of marmosets born in captivity^63^. As the marmoset monkeys typically give birth to dizygotic twins^23^, siblings from different litters are genetically as similar as same-litter siblings. The male monkey of the triplet (S1-1) was disowned by the parents on the third day and was hand-raised by an animal care-taker for the first 3 months. During this time, the monkey was placed in a small cage attached to the parents' home cage, allowing visual and auditory parental feedback for approximately 6–8 h per day. The other two members of the triplet (both females; S1-2 and S1-3) were raised by their parents until the end of the third month. After 3 months, we decided, in agreement with the veterinarians, to separate the two female marmosets from their parents to reunite them with their hand-raised sibling in a separate cage within the same animal facility. This gave us the unique possibility to integrate a hand-raised monkey into a stable social group and thus avoid having a hand-raised monkey be socially isolated. Therefore, all three monkeys had direct contact with their parents within the first 3 postnatal months. After separation, the triplet was continuously grouped together until and throughout the time of recordings. Cages were specifically arranged within the same animal room to avoid direct visual contact between animals from different cages. Therefore, the triplet had no visual contact with other marmosets during this time, but had continuous auditory contact with the other marmoset monkeys within the facility, including their parents. In addition, for all monkeys, physical and environmental conditions remained stable, such as temperature, humidity and the auditory feedback from the conspecifics housed within the same room. In contrast, the second set of marmosets (twins: S2-1 (male) and S2-2 (female)) were still grouped with the parents during the experiments of the present study. Therefore, both litters were held in similar conditions, which only differed in the presence and lack of direct parental feedback, respectively. This condition gave us the exceptional opportunity to compare the vocal behaviour of normally raised twins to the vocal behaviour of triplets that had parental feedback only during the first 3 months.

The marmoset monkeys in our colony were all born in captivity and are held in pairs or family groups. The colony room has a 12:12 day/night cycle with a temperature of approximately 27 °C and 45–55% relative humidity. The animals had *ad libitum* access to water and were fed on a daily basis with commercial pellets, curd cheese, fruits, vegetables, mealworms and locusts. Additional treats, such as marshmallow juice or fruit juice, were used as positive reinforcements to transfer the animals from their home cage to the experimental cage. All procedures were authorized by the national authority, the Regierungspräsidium Tübingen, Germany.

### Vocal recordings

We recorded the vocal behaviour of subadult marmosets when they were separated from their social group. For this purpose, animals were trained to enter an experimental cage (25 × 25 × 28 cm^3^), in which they were separated from their social group. During vocal recordings, animals that were raised by their parents for at least 3 months (S1-2, S1-3, S2-1 and S2-2) were visually and acoustically interacting with a pair of unrelated adult marmoset monkeys that were placed at a distance of approximately 1.5–2 m. The hand-raised monkey (S1-1) did not produce infant vocalizations towards other marmoset monkeys but produced them towards its main caregiver during infancy, an animal caretaker. Pygmy marmoset infants produce infant vocalizations especially in infant–caregiver interactions, as the caregivers are more likely to approach them when they do so^64^. Therefore, we recorded monkey S1-1 while having visual and acoustic contact with the animal caretaker, while all other monkeys had visual and acoustic contact with other marmoset monkeys. Vocalizations were recorded using a condenser microphone (Sennheiser MKH 8020 with preamplifier MZX 8000), which was placed in front of the small cage at a 10 cm distance, and digitized at a sampling frequency of 96 kHz via an A/D-interface (Roland UA-1010 OctaCapture). Daily sessions lasted 10–15 min each.

### Acoustic analysis

We recorded five daily sessions per individual monkey (5 monkeys, 25 sessions, 13,582 vocalizations). Call onsets and offsets were manually detected using the AVISOFT SASLab Pro 5.2 software (Avisoft Bioacoustics). Duration, peak frequency, inter-call interval and entropy values were extracted by using the same software. Duration was calculated as the difference between the end and the beginning of the vocalization. Peak frequency of a call was defined as the frequency with the maximum amplitude within the spectrum. Inter-call interval was defined as the difference between the onsets of two consecutive calls. Wiener entropy was used as a measure for how broadband the power spectrum of a specific sound is and was calculated as the logarithm of the ratio between the geometric and arithmetic means of the values of the power spectrum^16^,^18^. The signal was band-pass filtered between 5 and 15 kHz, as the great majority of marmoset calls fall into this range^24^,^65^. The spectrograms were calculated using FFT window of 1024 points, Hanning window and 50% overlap.

In the current study, we did not aim to classify calls within one call type into subtypes. We classified marmoset vocalizations into main groups already defined^22^,^24^. Calls were manually classified as cry, compound cry, trill, phee, peep, twitter, tsik and ekk calls based on their spectro-temporal profile and auditory playback. The eight call types showed a very defined and distinct profile and could be easily classified manually^16^,^17^,^24^,^65^. Cry is a broad-band call with the fundamental frequency (F0) around 600 Hz; compound cry is a combination of cry and another call regardless of the order; trill calls are defined by sinusoidal-like frequency modulation (FM) throughout the call; phee is a tone-like long call with F0 around 7–10 kHz; peeps are short duration calls with tone-like, sharply ascending or sharply descending FM; twitter is a short upward FM sweep; tsik is a broadband short call consisting of a linearly ascending FM sweep that merges directly into a sharply descending linear FM sweep. Ekk is a short call that is defined as one of the lowest frequency marmoset calls. Trill–phees are a combination of two call types, trill and phee, and were classified into the call type that was predominantly present within the call.

### Statistical analyses

Our group of five animals was tested by a two-sampled χ^2^ test to reveal differences in the distribution of different call types and call transitions. For call-type distribution, we looked at the differences in the distribution of eight call types within the group of our five animals. For call transition, we tested for the differences in distribution of all occurred call transitions between animals. We performed Kruskal–Wallis tests to reveal differences in call frequency and inter-call intervals between the animals. For inter-call interval, we focussed on the range between 0 and 2 s (11,370 calls), as changes in inter-call intervals were most prominent below 2 s. In all performed tests, significance was tested at an alpha=0.05 level. When significant differences were found in one of the above-mentioned distributions, we performed *post hoc* clustering analyses using weighted linkage (proximity matrix with Spearman's distance)^66^. Using this approach, we were able to subdivide our group of five animals into distinct clusters. Statistical analysis was performed using MATLAB (MathWorks, Natick, MA, USA).

### Data availability statement

The data that support the findings of this study are available from the corresponding author on request.

## Additional information

**How to cite this article:** Gultekin Y. B. & Hage S. R. Limiting parental feedback disrupts vocal development in marmoset monkeys. *Nat. Commun.* **8**, 14046 doi: 10.1038/ncomms14046 (2017).

**Publisher's note:** Springer Nature remains neutral with regard to jurisdictional claims in published maps and institutional affiliations.
